# A novel serum metabolomic panel distinguishes IgG4‐related sclerosing cholangitis from primary sclerosing cholangitis

**DOI:** 10.1111/liv.15192

**Published:** 2022-02-21

**Authors:** Daniel E. Radford‐Smith, Emmanuel A. Selvaraj, Rory Peters, Michael Orrell, Jonathan Bolon, Daniel C. Anthony, Michael Pavlides, Kate Lynch, Alessandra Geremia, Adam Bailey, Emma L. Culver, Fay Probert

**Affiliations:** ^1^ Department of Pharmacology University of Oxford Oxford UK; ^2^ Department of Chemistry University of Oxford Oxford UK; ^3^ Translational Gastroenterology Unit, Nuffield Department of Medicine University of Oxford Oxford UK; ^4^ Oxford Centre for Clinical Magnetic Resonance Research (OCMR), Radcliffe Department of Medicine University of Oxford Oxford UK; ^5^ NIHR Oxford Biomedical Research Centre University of Oxford and Oxford University Hospitals NHS Foundation Trust Oxford UK

**Keywords:** biomarkers, cholangitis, diagnosis, immunoglobulin G4‐related disease, metabolomics, sclerosing

## Abstract

**Background & Aims:**

Primary sclerosing cholangitis (PSC) and IgG4‐related sclerosing cholangitis (IgG4‐SC) are chronic fibro‐inflammatory immune‐mediated hepatobiliary conditions that are challenging to distinguish in a clinical setting. Accurate non‐invasive biomarkers for discriminating PSC and IgG4‐SC are important to ensure a correct diagnosis, prompt therapy and adequate cancer surveillance.

**Methods:**

We performed nuclear magnetic resonance (NMR)‐based metabolomic profiling using serum samples collected prospectively from patients with PSC (*n* = 100), IgG4‐SC (*n* = 23) and healthy controls (HC; *n* = 16).

**Results:**

Multivariate analysis of the serum metabolome discriminated PSC from IgG4‐SC with greater accuracy (AUC 0.95 [95%CI 0.90–0.98]) than IgG4 titre (AUC 0.87 [95%CI 0.79–0.94]). When inflammatory bowel disease (IBD) was excluded as a comorbid condition (IgG4‐SC *n* = 20, PSC *n* = 22), the diagnostic AUC increased to 1.0, suggesting that the metabolome differences identified are not a result of the increased prevalence of IBD in PSC relative to IgG4‐SC patients. Serum lactate (*p* < .0001), glucose (*p* < .01) and glutamine (*p* < .01) metabolites were increased in IgG4‐related disease (IgG4‐RD) and IgG4‐SC individuals compared to PSC, whereas mobile choline (*p* < .05), 3‐hydroxybutyric acid (*p* < .01) and ‐CH_3_ lipoprotein resonances (*p* < .01) were decreased.

**Conclusions:**

Taken together, serum metabolomic profiling has the potential to be incorporated as a diagnostic criterion, independent of IgG4 titre, to improve the diagnosis of IgG4‐RD and help distinguish IgG4‐SC from PSC.

**Abbreviations**
AIP: autoimmune pancreatitis
AUC: area under curve
BCR: B‐cell receptor
CI: Confidence interval
HC: Healthy control
IBD: Inflammatory bowel disease
IgG4‐RD: Immunoglobulin G4‐related disease
IgG4‐SC: Immunoglobulin G4‐related sclerosing cholangitis
NMR: Nuclear magnetic resonance
OPLS‐DA: Orthogonal partial least squares discriminant analysis
PSC: Primary sclerosing cholangitis
ROC: Receiver operating characteristic
sIgG4: serum IgG4
UDCA: Ursodeoxycholic acid
VIP: Variable importance in projection

## 1 INTRODUCTION

Primary sclerosing cholangitis (PSC) and IgG4‐related sclerosing cholangitis (IgG4‐SC) are progressive fibro‐inflammatory immune‐mediated conditions associated with organ dysfunction and failure, malignancy and mortality. PSC and IgG4‐SC share commonalities such as male preponderance, clinical presentation with obstructive jaundice, cholestatic liver biochemistry and bile duct stenoses on cholangiogram.^1^ However, whilst corticosteroids improve disease in patients with IgG4‐SC, they are ineffective in classical PSC and may be detrimental in those with advanced disease. Currently, there is no medical therapy for patients with PSC and liver transplantation remains the only cure, with a risk of relapse in the graft. IgG4‐SC/autoimmune pancreatitis has an increased risk of all‐cause malignancy (odds ratio 2.2), whilst PSC has a substantial risk of colorectal and hepatobiliary malignancy (odds ratio 4.5), with established surveillance performed in the latter.^2^ With prompt diagnosis and therapy, IgG4‐SC has a more favourable outcome than PSC, associated with a lower risk of developing cirrhosis and cholangiocarcinoma. Thus, the early and accurate distinction between IgG4‐SC and PSC is essential to ensure appropriate treatment and cancer surveillance is performed.

To date, the rare and heterogeneous nature of these conditions has hampered the discovery of reliable biomarkers distinguishing PSC from IgG4‐SC. Measurement of serum IgG4 (sIgG4) level is a key part of the diagnostic criteria for IgG4‐RD, however, it is limited by poor sensitivity and specificity for the diagnosis of IgG4‐SC (20% have a normal serum IgG4 level).^3^ Additionally, it can be raised in 10‐25% of patients with PSC, where it is associated with disease progression and a worse outcome.^1^ A ratio of serum IgG4/IgG1 was reported to improve discrimination between PSC and IgG4‐SC in those with a moderately elevated sIgG4 level (1.4‐2.8 g/L).^4^ In another study, IgG4+ B‐cell receptor (BCR) clones were found to predominate in both the peripheral blood and affected tissue of patients with IgG4‐SC, but not in patients with PSC.^5^ Subsequently, a quantitative PCR assay was designed to measure IgG4+ BCR mRNA molecules (as a proxy of the extent of IgG4+ B‐cell clonal expansion) in peripheral blood. An optimal cut‐off diagnosed IgG4‐SC, as opposed to PSC and biliary/pancreatic malignancies, with an area under the curve (AUC) value of 0.99.^6^

Metabolomics, the measurement of all small molecular intermediates, or metabolites, in a biological sample, has emerged as a powerful method of profiling disease phenotypes.^7^ Alterations of the metabolome have been described for several autoimmune and inflammatory conditions. Changes in nuclear magnetic resonance (NMR)‐detectable *N*‐acetylated‐glycoproteins and serum lipoprotein species are known to be associated with the acute phase response and chronic inflammation, respectively. We have previously demonstrated that decreases in serum lipoproteins along with increased glucose and valine concentrations are associated with disease severity and inflammation in patients with ulcerative colitis.^8^ We have also shown that NMR metabolomics analysis of blood is able to distinguish between cell‐mediated and antibody‐mediated inflammation of the central nervous system.^9^ In this study, we aimed to assess whether the same blood‐based NMR metabolomics method could be used to distinguish between PSC and IgG4‐SC.

Herein, we performed the first untargeted serum NMR‐based metabolomics in a cohort of 139 participants comprised of PSC, IgG4‐SC, IgG4‐RD without hepatobiliary involvement, and healthy controls (HCs). Metabolome data were investigated using multivariate statistical techniques with the aims of distinguishing PSC from IgG4‐SC and exploring the molecular mechanisms underlying these conditions and compared to those of the control cohort to provide ‘healthy’ reference ranges.

## 2 PATIENTS AND METHODS

### 2.1 Study participants

The study cohort comprised individuals who had a serum sample available which was frozen at collection and then thawed only on the day of NMR spectroscopy (*n* = 155). Patients with IgG4‐RD (*n* = 39, 23 with biliary disease), PSC (*n* = 100) and HC (*n* = 16) were prospectively recruited between 2011 and 2018 from the outpatient clinics of Oxford University Hospitals NHS Foundation Trust, a tertiary referral centre for IgG4‐RD and PSC. Patients enrolled in this study had clinical data and serum samples collected and were then prospectively followed up. Enrolled study participants provided written informed consent. The study was approved by the Research Ethics Committee, Oxfordshire (ref:10/H0604/51, ref:16/YH/0247, and ref:18/SC/0367) and registered on the NIHR UK portfolio (10776). It was conducted in accordance with the study protocol and the principles of the Declaration of Helsinki (2008), and the International Conference on Harmonization (ICH) Good Clinical Practices standards.

### 2.2 Patient and Public Involvement Statement

EC is on the Steering Committee for the British Association for the Study of the Liver (BASL), Immune‐Mediated Liver Disease (IMLD), Special Interest Group (SIG), UK PSC and UK IgG4. Patient representatives with PSC and IgG4‐SC were able to provide feedback on the study concept and design of the experiment. A lay summary of the results of this study will be made available on the Oxford NDM, PSC Support and IgG4‐RD websites.

### 2.3 Diagnostic criteria

The diagnosis of IgG4‐RD was made using the HISORt criteria (2006/7) for IgG4‐related pancreatic and biliary disease,^10^ and the Japanese Comprehensive Diagnostic Criteria (2011) for systemic disease.^11^ Organ involvement included the bile ducts (23), pancreas (23), salivary glands (9), lacrimal glands (5), lymph nodes (5), lung (6), kidney (5), retroperitoneum (3) and aorta (1). The diagnosis of PSC was made in accordance with the EASL guidelines for cholestatic liver disease.^12^ HC had normal liver biochemistry, no previous self‐reported history of liver or biliary disease or intervention, no inflammatory bowel disease (IBD) or diabetes, alcohol consumption within the current recommended limit and body mass index not greater than 25 kg/m^2^.

For details on clinical data, sample collection and processing, NMR spectroscopy and data preprocessing, refer to supplementary methods.

### 2.4 Statistical analysis

Multivariate orthogonal partial least squares discriminant analysis (OPLS‐DA) was performed in R software (R foundation for statistical computing, Vienna, Austria) (R Development Core Team, 2019) using in‐house R scripts and the ropls package.^13^ All models were validated on independent (unseen) test data using an external 10‐fold cross‐validation strategy with repetition coupled with permutation testing as previously described^8^, ^14^ (Figure S1). If, after cross‐validation with repetition, the model performed significantly better than random chance (determined using the two‐sided Kolmogorov Smirnov test), a single OPLS‐DA of all the data was used to report a final diagnostic accuracy, sensitivity, specificity, positive predictive value and AUC value using the receiver operating characteristic (ROC) curve analysis and Youden index. Variables responsible for the observed class separation were extracted by inspection of the average variable importance (VIP) scores.

All univariate and ROC analysis were performed in R software and GraphPad Prism 9.0 (GraphPad Software). For clinical chemistry and baseline characteristics, the Mann Whitney *U* test was applied, comparing PSC and IgG4‐RD. Chi‐squared tests were used for categorical variables. To compare individual metabolite differences between PSC, IgG4‐RD and HC, Brown‐Forsythe and Welch ANOVA with Dunnet’s T3 post hoc comparisons was used. A multiple comparisons correction (Bonferroni) was applied throughout. Two‐tailed *p* < .05 were considered statistically significant. ROC, AUC, 95% confidence intervals (CI) and optimal thresholds for diagnosis were calculated using the pROC package.^15^

## 3 RESULTS

### 3.1 Cohort characteristics

Serum NMR spectra from 39 patients with IgG4‐RD (IgG4‐SC *n* = 23), 100 patients with PSC (large‐duct *n* = 81, small‐duct *n* = 19) and 16 HC were included in the analysis. The baseline characteristics of the 139 patients are summarized in Table 1. HC baseline characteristics are included in Table S1.

**TABLE 1:** Baseline characteristics of patients with PSC and IgG4‐RD

	PSC (*n* = 100)	IgG4‐RD (*n* = 39)	*p*‐value
Age (years)^b^	45.0±19.0 (12‐84)	64.9±12.6 (33‐83)	<.0001
Sex (% female)	38.0	15.8	.11
Disease duration (years)^a^	5.0, 1.0‐10.0 (0‐22)	1.0, 0.0‐6.0 (0‐11)	.09
IBD (%)	78.0	10.5	<.0001
Current UDCA use (%)	51.0	2.7	<.0001
Diabetes (%)	6.0	20.5	.08
IgG4 titre (g/L)^a^	0.50, 0.26‐0.81 (0.03‐67.40)	2.70, 1.22‐5.13 (0.12‐17.20)	<.0001
ALT (IU/L)^a^	38.5, 23.8‐82.8 (3‐2683)	25.0, 17.0‐41.8 (8‐1034)	.21
ALP (IU/L)^a^	210.5, 113.0‐372.0 (42‐1316)	157.0, 112.3‐208.8 (33‐578)	.33
Albumin (g/L)^b^	38.52 ± 5.58 (23‐50)	40.29 ± 5.32 (26‐49)	>.99
Bilirubin (μmol/L)^a^	10.5, 8.0‐20.0 (3‐286)	9.0, 8.0‐16.0 (4‐203)	>.99
IgG titre (g/L)^a^	13.4, 11.2‐17.2 (7.2‐23.7)	12.8, 10.6‐15.7 (4.9‐34.1)	>.99

Abbreviations: ALP, alkaline phosphatase; ALT, alanine aminotransferase; IBD, inflammatory bowel disease; IgG4‐RD, IgG4‐sclerosing cholangitis; PSC, primary sclerosing cholangitis; UDCA, ursodeoxycholic acid.

^a^ Median, IQR (range).

^b^ Mean ± SD (range).

### 3.2 Serum IgG4 titre distinguishes IgG4‐RD and IgG4‐SC from PSC with high sensitivity but low specificity

**FIGURE 1:**
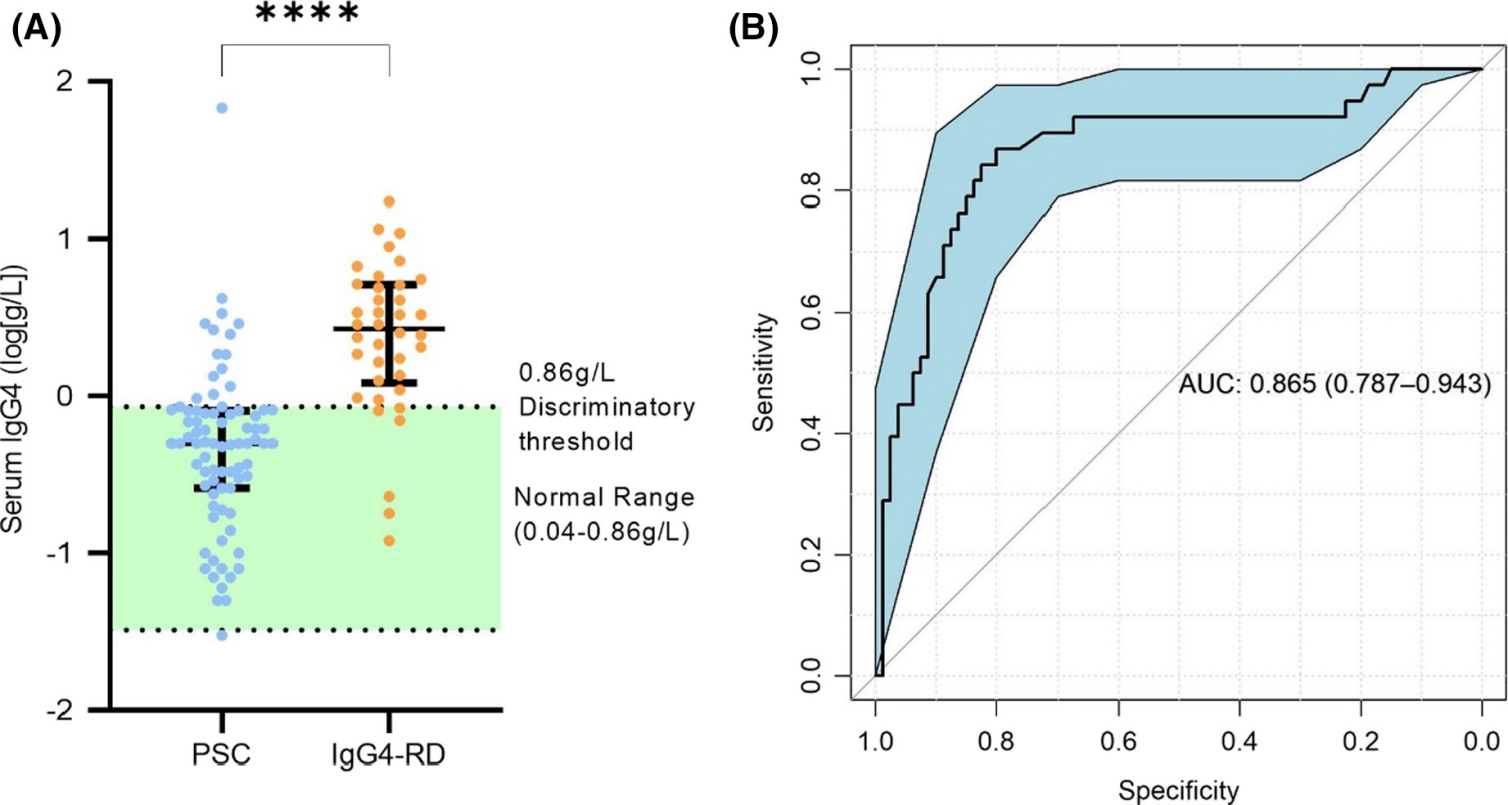
Serum IgG4 titre discriminates between IgG4‐related disease (IgG4‐RD) and primary sclerosing cholangitis (PSC) with high sensitivity but low specificity. (A) Patients with IgG4‐RD had elevated serum IgG4 titres (Mann Whitney *U* test, *p* < .0001). Median ± interquartile range showing all points. While HC serum IgG4 was not measured, the normal range (.04–0.86 g/L) is indicated in green. (B) ROC curve for IgG4 titre classifying PSC vs IgG4‐RD. ROC curves show AUC ± 95% confidence intervals. *N* = 80 (PSC) and 38 (IgG4‐RD). ****Mann Whitney U test, *p* < .0001

Firstly, we assessed the diagnostic specificity of serum IgG4 titres in distinguishing patients with hepatobiliary disease caused by IgG4‐SC and PSC. Data pertaining to serum IgG4 level at diagnosis was available for 38/39 patients with IgG4‐RD (22/23 IgG4‐SC) and 80/100 patients with PSC. As expected, IgG4 titre discriminated between PSC and IgG4‐RD with a high area under curve (AUC) value of 0.87 (95%CI 0.79‐0.94; Figure 1A). Our institutional threshold for an elevated serum IgG4 titre (>0.86 g/L) is equal to the optimum threshold as determined by the Youden index for this cohort (Figure 1B), leading to a high sensitivity of 91.7%, although specificity was low at 69.6%. As shown in the confusion matrix (Table S2), six of the 38 patients with IgG4‐RD (15.8%) had a titre within the normal range (<0.86 g/L) whereas 14 of the 80 patients with PSC (17.5%) had a high IgG4 titre above the normal range (>0.86 g/L). This led to an overall positive predictive value of 82.5%. IgG4 titre remained highly discriminatory when IgG4‐SC was compared to PSC (ROC AUC 0.89 [95%CI 0.81‐0.97], Figure S2A,B). Despite a high sensitivity of 97.1%, the specificity remained low, at 58.8%. This can be seen in the confusion matrix (Table S2) as, although 20 of 22 IgG4‐SC individuals had a high serum IgG4 titre, 14 PSC individuals were also in this category. When individuals with IBD were excluded, IgG4 titre remained accurate in classifying IgG4‐SC from PSC (ROC AUC 0.92 [95%CI 0.85‐1.0], Figure S2C,D). The addition of other routine clinical chemistry parameters (shown in Table 1) did not afford any further increase in diagnostic accuracy when discriminating IgG4‐RD (Figure S3A,B) or IgG4‐SC (Figure S3C,D) from PSC. This confirmed that a diagnostic algorithm, superior to serum IgG4 titre levels, could *not* be generated from the combination of pre‐existing standard biochemical tests.

### 3.3 Serum metabolomics distinguishes between IgG4‐SC and PSC with greater accuracy and specificity than IgG4 titre

**FIGURE 2:**
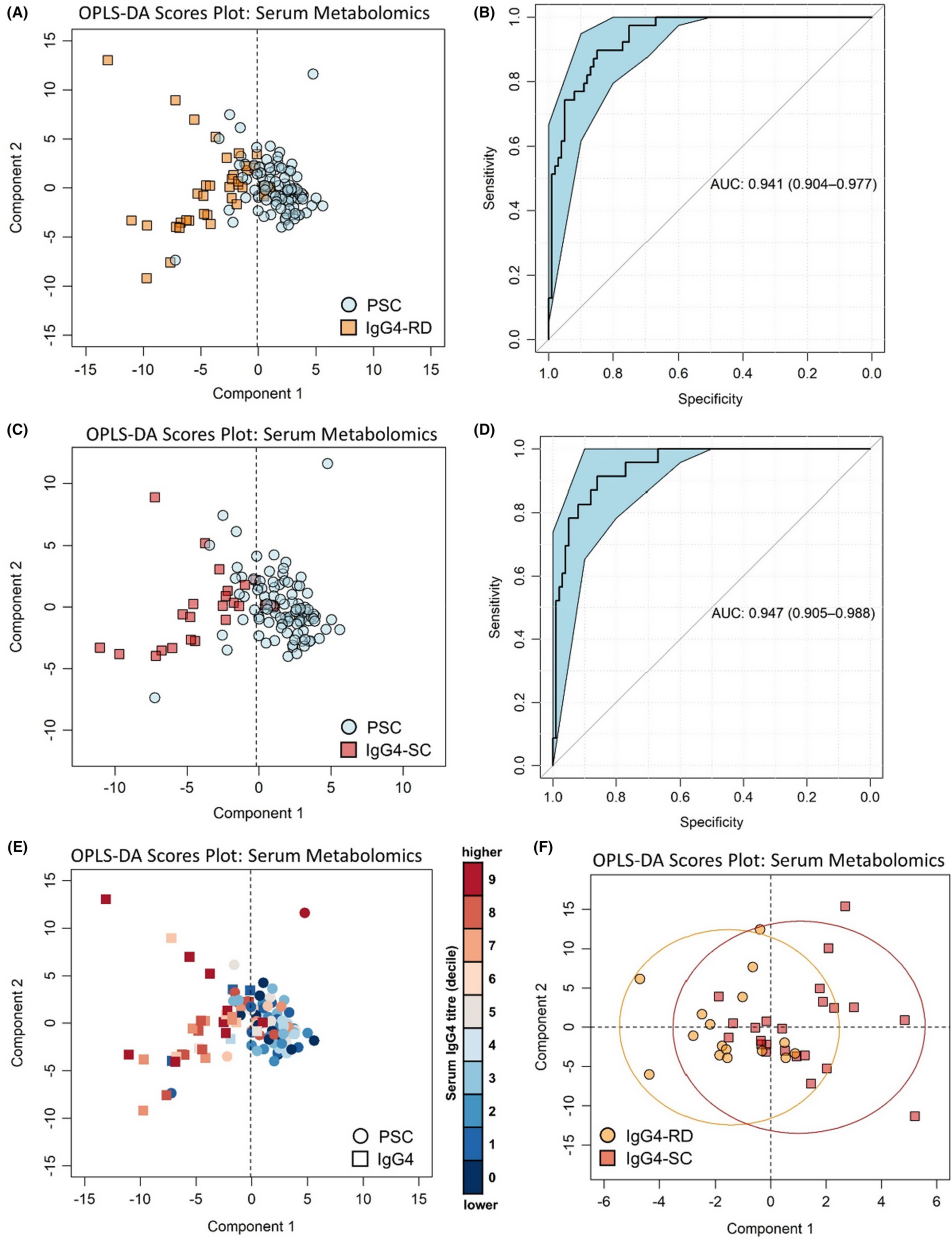
Multivariate analysis of serum metabolites discriminates PSC from IgG4‐RD with 86% diagnostic accuracy. PSC vs IgG4‐RD disease (a) OPLS‐DA scores plot and (b) corresponding ROC curve. PSC vs IgG4‐SC (c) OPLS‐DA scores plot and (d) corresponding ROC curve. (e) OPLS‐DA scores plot of PSC vs IgG4 disease coloured by serum IgG4 titre. (f) OPLS‐DA scores plot of IgG4‐RD (excl. IgG4‐SC) vs IgG4‐SC. ROC curves show AUC ± 95% confidence intervals. *N* = 100 (PSC), 23 (IgG4‐SC), 16 (IgG4‐RD excl. IgG4‐SC)

Multivariate analysis (OPLS‐DA) of >100 serum metabolites, simultaneously quantified by ^1^H‐NMR spectroscopy, was used to generate models distinguishing 39 patients with IgG4‐RD from 100 patients with PSC (Figure S1). The clear clustering observed in the resulting OPLS‐DA scores plot illustrates the distinct serum metabolic profiles intrinsic to IgG4‐RD and PSC (Figure 2A). External cross‐validation coupled with permutation testing confirmed that the model performed significantly better than a random null distribution and was not a result of over‐fitting the data. IgG4‐RD was distinguished from PSC with an accuracy of 87%, AUC of 0.94 (95%CI 0.90‐0.98), sensitivity of 96% and specificity of 70% (Figure 2B). The positive predictive value was 85%. The equal dispersion of IgG4‐RD and IgG4‐SC patients on the left side of the scores plot (Figure 2A) indicate that IgG4‐SC and IgG4‐RD are much more similar to each other than compared to PSC. Indeed, no significant difference in the serum metabolome of IgG4‐RD (*n* = 16) and IgG4‐SC (*n* = 23) was identified by OPLS‐DA (Figure 2F). When IgG4‐SC patients were specifically compared to PSC patients (Figure 2C), the accuracy was 87.0%, the AUC was 0.95 (95%CI 0.91‐0.99; Figure 2D) and the positive predictive value was 88%. The main OPLS‐DA model represented in Figure 2A correctly identified PSC patients with high serum IgG4 levels (Figure 2E) and was not affected by ursodeoxycholic acid (UDCA) therapy in the PSC patients (Figure S4). Similarly, corticosteroid and conventional immunosuppressive agents in the IgG4 patients did not contribute to the discriminatory accuracy of the main OPLS‐DA model. Individuals with IgG4 disease naïve to treatment did not cluster with PSC individuals who were also treatment‐naïve (Figure S5).

As a direct comparison to IgG4 titre, another ROC curve was generated using only those individuals with IgG4 titre data (*n* = 80 PSC, *n* = 38 IgG4). Classification using the serum metabolomic data reduces the proportion of IgG4 individuals incorrectly identified as ‘PSC’ by antibody titre from 6 (Table S2) to 4 (Table S3). The proportion of PSC individuals incorrectly identified as IgG4 by antibody titre was also reduced, from 14 (Table S2) to 8 (Table S3). When only IgG4‐SC patients were classified against PSC patients with IgG4 titre, the sensitivity was similar to antibody titre (97.3% compared to 97.1%); indeed, the same number of IgG4‐SC patients were correctly classified (20/22; Table S3). Because of the improvement in correctly classifying PSC patients with high IgG4 titres (Figure 2E), the specificity of serum metabolomics, compared to IgG4 titre alone, is greatly improved for distinguishing IgG4‐SC (71.4% compared to 58.8%).

### 3.4 Metabolomics analysis reveals a distinct serum metabolite signature in IgG4‐SC patients compared to PSC patients which is independent of IgG4 titre

**FIGURE 3:**
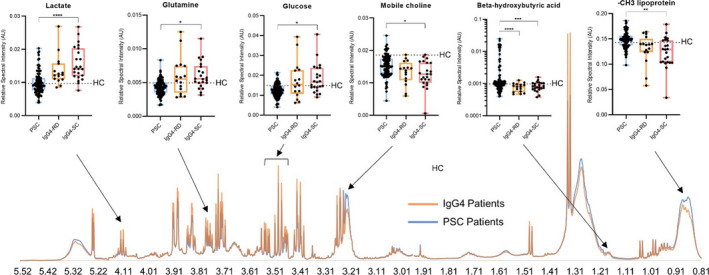
Altered metabolite concentrations indicate a molecular signature of IgG4‐RD compared to PSC. NMR spectra showing median intensity of IgG4 (orange) and PSC (blue) individuals, and box plots of key metabolites. Whiskers indicate min/max values showing all datapoints. Welch’s one‐way ANOVA followed by Dunnett’s post hoc comparisons and Bonferroni’s correction for multiple tests: Lactate (*p* < .0001), glutamine (*p* < .05) and glucose (*p* < .05) were higher in IgG4‐SC patients (*n* = 23) than in PSC patients (*n* = 100), while ‐CH_3_ lipoprotein (*p* < .01), mobile choline (*p* < .05), and 3‐hydroxybutyric acid (*p* < .001) were decreased. Similar trends in metabolite concentrations were observed in IgG4‐RD patients (*n* = 16) for lactate (*p* = .055), glutamine (*p* = .084), glucose (*p* = .12) and ‐CH_3_ lipoprotein (*p* = .051), while 3‐hydroxybutyric acid was also significantly decreased (*p* < .0001). The dotted line in the univariate plots represents the average healthy control value. *p*‐values < .05, .01, .001 and .0001 are represented by *, **, *** and ****, respectively

The serum metabolites that contributed most to the variability between IgG4‐RD and PSC patients, as ranked by VIP score from the OPLS‐DA projection, were investigated further (Table 2). A separate analysis of IgG4‐SC and PSC yielded a similar shortlist of important metabolites, which was expected given there was no difference in the serum metabolome between IgG4‐RD and IgG4‐SC (Figures 2F and 3). Serum lactate, glucose and glutamine were found to be increased in the sera of individuals with IgG4‐RD (including those with IgG4‐SC), relative to those with PSC (Figure 3). Conversely, serum lipoprotein resonances, especially chemical shift regions corresponding to smaller, higher density lipoprotein particles, were found to be significantly lower in IgG4‐RD (including those with IgG4‐SC) compared to PSC (Figure 3). The metabolome profiles of the control cohort were investigated to provide ‘healthy’ reference ranges for metabolite markers. Both IgG4‐RD and PSC were independently distinct from HC (Figure S6A‐D) providing further evidence that the metabolites identified are associated with the distinct pathogeneses of these diseases. The serum metabolome of those with large duct compared to those with small duct PSC could not be accurately distinguished (Figure S7A,B).

**TABLE 2:** Summary of discriminatory metabolites

Metabolite	PSC vs HC	IgG4‐RD vs HC	IgG4‐RD vs PSC
Lactate	1.0 (.99)	**1.5 (<.0001)**	**1.5 (<.0001)**
Glutamine	.86 (.18)	1.26 (.21)	**1.47 (.0036)**
Glucose	.93 (.28)	1.29 (.32)	**1.38 (.0072)**
‐CH_3_ Lipoprotein	1.07 (.99)	**.93 (.048)**	**.87 (.0012)**
Mobile choline	**.79 (.0012)**	**.68 (<.0001)**	**.87 (.015)**
3‐hydroxybutyric acid	**2.98 (.0036)**	.86 (.99)	**.29 (.0012)**

*Note:* Data showing fold change (*p*‐value). Metabolites with *p*‐value < .05 are highlighted in bold.

Abbreviations: HC, healthy controls; IgG4‐RD, IgG4‐related disease; PSC, primary sclerosing cholangitis.

### 3.5 The distinct serum metabolic profiles of PSC and IgG4‐SC are not obscured by the much higher prevalence of comorbid IBD in patients with PSC

**FIGURE 4:**
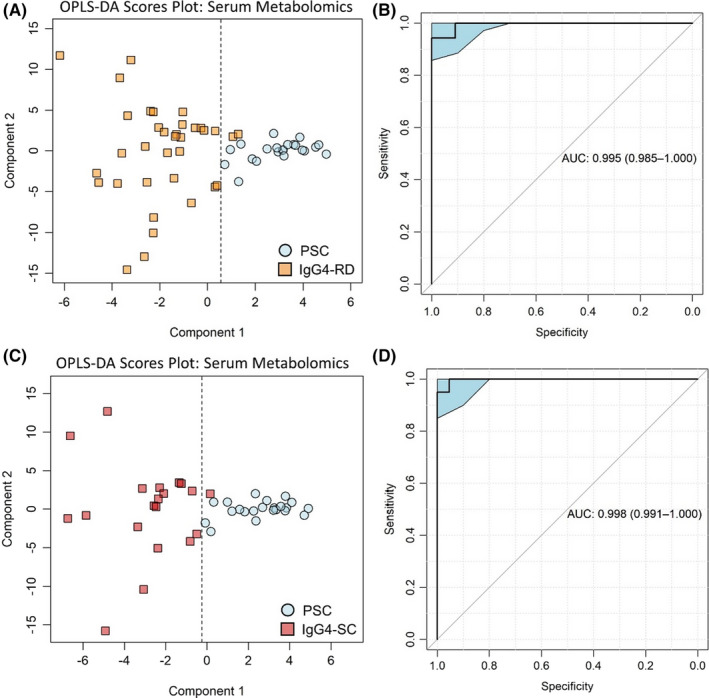
The distinct serum metabolic profiles of PSC and IgG4‐SC are not obscured by the much higher prevalence of comorbid inflammatory bowel disease (IBD) in PSC patients. PSC vs IgG4‐RD (a) OPLS‐DA scores plot and (b) corresponding ROC curve. PSC vs IgG4‐SC (c) OPLS‐DA scores plot and (d) corresponding ROC curve. Models were built using serum metabolomic data only and excluded patients with IBD. ROC curves show AUC ±95% confidence intervals. *N* = 22 (PSC), 20 (IgG4‐SC), 15 (IgG4‐RD excl. IgG4‐SC)

The presence of IBD is common in individuals with PSC, but infrequent in IgG4‐SC. In this cohort, 78% of PSC patients had co‐existing IBD (predominantly ulcerative colitis), whereas only 10% of IgG4‐SC had IBD (indeterminate colitis). Given this discrepancy, it is important to confirm that the metabolites which discriminate between IgG4‐SC and PSC are not confounded by the IBD prevalence. To account for this, new OPLS‐DA models were generated, excluding all patients with IBD co‐morbidity. There was a clear separation in the serum metabolic profiles of PSC (*n* = 22) and IgG4‐RD (*n* = 35) remained significant when patients with IBD were removed (Figure 4A). The accuracy of the OPLS‐DA model was 97%, with a sensitivity of 92%, specificity of 100% and positive predictive value of 100%. The AUC of the discriminatory ROC curve was 1.0 (95% CI 0.99–1.0; Figure 4B). IgG4‐SC was also compared specifically to PSC in the absence of IBD (Figure 4C). Here, the accuracy was 98%, with a sensitivity of 100%, specificity of 95% and positive predictive value of 96%. The AUC of the discriminatory ROC curve was 1.0 (95% CI 0.99–1.0; Figure 4D). The metabolites contributing to these models were similar to those that discriminated IgG4‐RD and IgG4‐SC from PSC in the entire cohort. This indicates that the unique metabolic signatures of IgG4‐SC and PSC can be identified amidst other autoimmune, inflammatory diseases.

### 3.6 The distinct metabolic signatures of IgG4‐SC and PSC are robust to demographic differences

**FIGURE 5:**
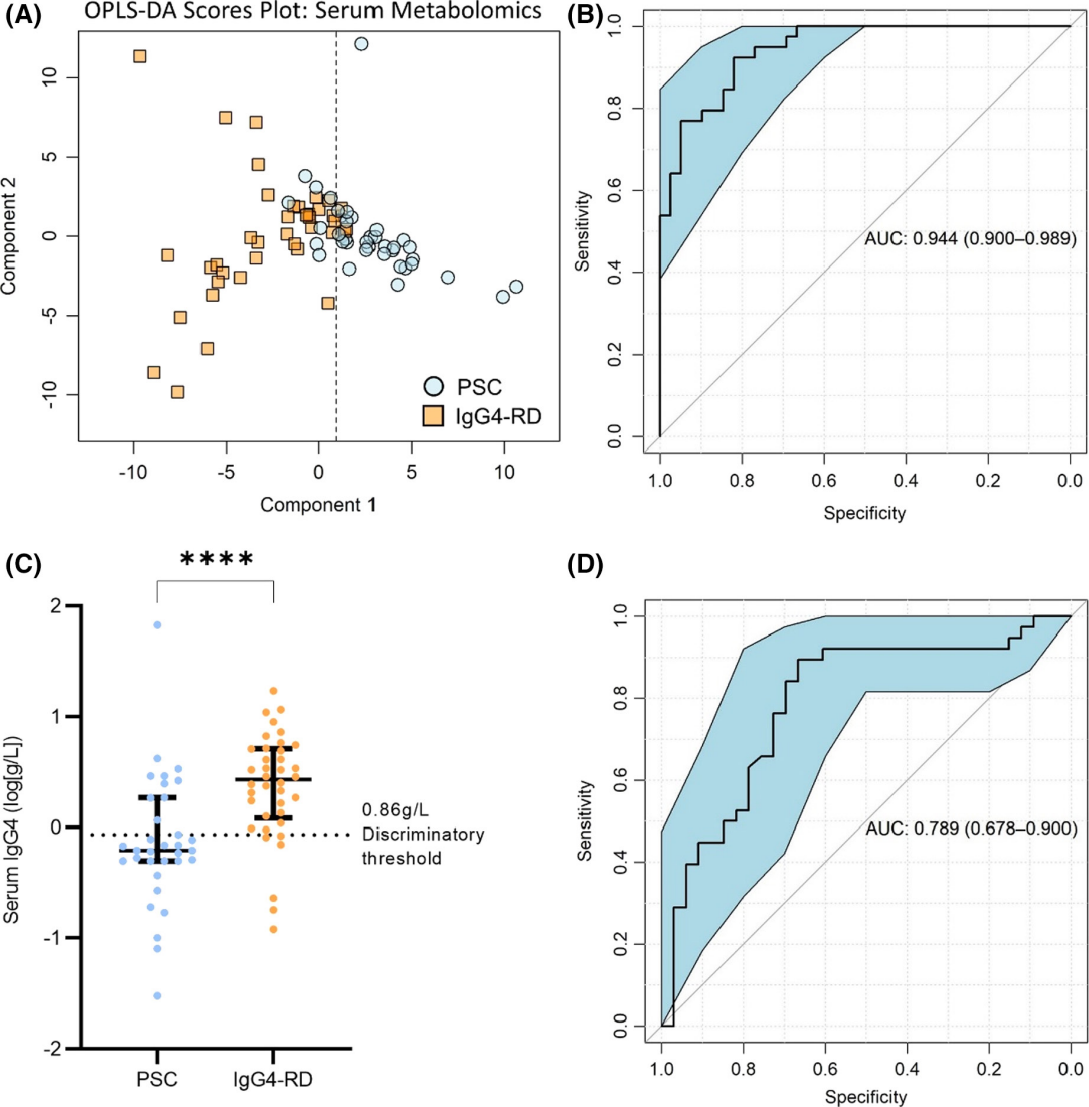
Serum metabolomics remains more specific than IgG4 titre when discriminating IgG4‐RD from PSC in age and sex‐matched patient subgroup. (a) Boxplot of serum IgG4 antibodies in IgG4 disease and PSC patients in age and sex‐matched subgroup (Kolmogorov Smirnov, *p* < .0001). Median ± interquartile range showing all points. (b) ROC curve for IgG4 titre classifying PSC vs IgG4 disease in matched subgroup. (c) Multivariate scores plot of PSC vs IgG4 disease using serum metabolomic data. (d) ROC curve for serum metabolomic data, classifying PSC and IgG4 disease in the matched cohort. ROC curves show AUC ±95% confidence intervals. *N* = 39 (PSC) and 39 (all IgG4‐RD). ****p‐values < .0001

Patients with IgG4‐RD were older^16^, ^17^ at the time of sampling compared with the PSC population (Table 1). Because the profile of the serum metabolome changes with age independent of disease,^18^ it was necessary to verify the metabolomic model, controlling for age at sampling. From the PSC cohort (*n* = 100), age, disease duration and sex‐matched subset (*n* = 39) were selected. Apart from excluding all PSC patients outside the age range of the IgG4 cohort, the selection of the age‐matched PSC subset was random. The serum metabolomic OPLS‐DA model remained highly discriminatory (Figure 5A) with an accuracy of 87% and a positive predictive value of 82%. The ROC AUC for this model was 0.94 (95% CI 0.90‐0.99; Figure 5B). In comparison, IgG4 titre from the same subset of patients (Figure 5C) had an AUC of 0.79 (95%CI 0.68‐0.90; Figure 5D) and a positive predictive value of 70%. Metabolites contributing to this model were also representative of the previous models, though glutamine level had an increased weighting in the model relative to lipoproteins, glucose and lactate.

### 3.7 A panel of four serum metabolites retains a high discriminatory capacity

**FIGURE 6:**
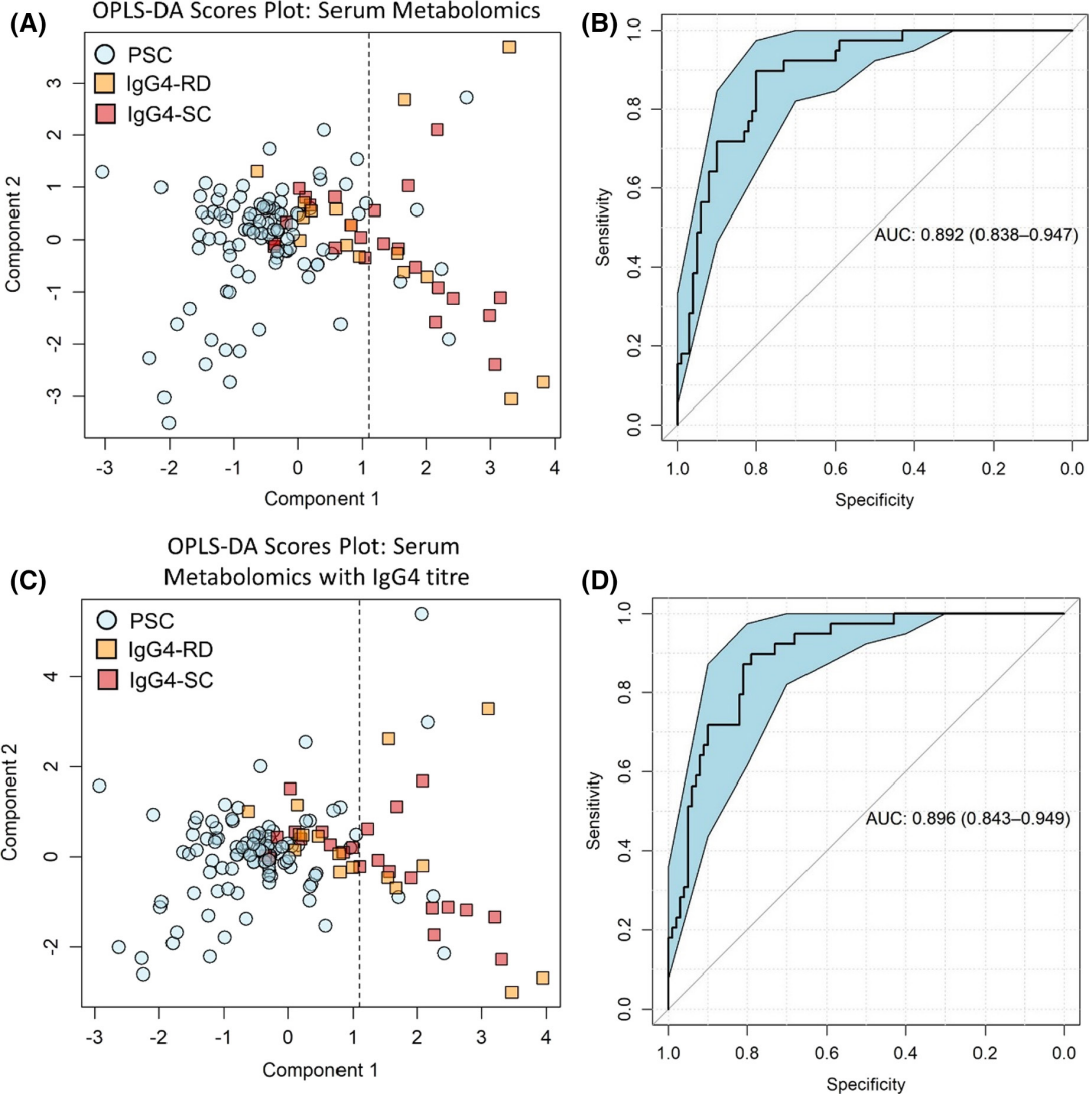
A panel of four serum metabolites retains a high discriminatory capacity. Multivariate scores plot of (a) PSC vs IgG4‐RD and (c) PSC vs IgG4‐SC using only spectral peaks pertaining to beta‐hydroxy butyric acid, lactate, mobile choline and glutamine. (b) ROC curve generated from the three metabolites, classifying PSC vs IgG4‐RD and (d) classifying PSC vs IgG4‐SC. ROC curves show AUC ±95% confidence intervals. *N* = 100 (PSC) and 39 (all IgG4‐RD)

Whilst ^1^H‐NMR allows for the simultaneous global measurement of metabolite concentrations in patient sera, most did not yield an association between concentration and PSC or IgG4 disease. We show that the multivariate analysis of four metabolites (3‐hydroxybutyric acid, lactate, glutamine and mobile choline) affords a discriminatory accuracy of 82% (Figure 6A), sensitivity of 83%, specificity of 79% and positive predictive value of 95%, with an AUC value of 0.89 (95% CI 0.84–0.95; Figure 6B). Including IgG4 titre in the same model did not significantly increase the accuracy of the model to 83% (Figure 6C). Model specificity was 80% and sensitivity 83%, and the overall AUC was increased to 0.90 (95%CI 0.84–0.95; Figure 6D).

## 4 DISCUSSION

Distinguishing PSC from IgG4‐RD with hepatobiliary involvement (IgG4‐SC) is challenging.^1^ In this study, sIgG4 titre diagnosed IgG4‐RD, as opposed to PSC, with high sensitivity but low specificity, whilst the addition of routine clinical chemistry afforded no increase in diagnostic accuracy. Accordingly, we used NMR metabolomics and OPLS‐DA to investigate the serum metabolome of these patients. We identified distinct serum metabolic signatures of IgG4‐RD and PSC, which allowed the accurate diagnosis of IgG4‐SC from PSC with much greater specificity than sIgG4 titre. Furthermore, post hoc investigation of the metabolite profiles provided novel insights into the distinct molecular mechanisms driving IgG4‐RD and PSC.

To date, only two studies have investigated the metabolome in patients with PSC and there are no previous studies which have used NMR metabolomics methods. While serum metabolomics of portal blood by mass spectrometry showed altered composition compared to cirrhosis and HCs,^19^ a comparison to IgG4‐SC has never been reported. An integrated analysis of the faecal metabolome and microbiome in PSC and IgG4‐SC revealed distinct disease profiles.^20^ Despite a high level of microbe‐metabolite correlation, faecal metabolites performed better at predicting disease status than microbial taxa. In contrast, the study presented here, is the first to apply blood‐based NMR metabolomics to directly compare PSC with IgG4‐SC.

Certain key metabolites discriminating IgG4‐RD from PSC were similar to those discriminating IgG4‐RD from HC individuals. For example, serum lactate was found to be elevated in those with IgG4‐RD compared to PSC, where patients with PSC had similar serum lactate levels compared to HC individuals. While no studies have previously investigated serum metabolic differences between PSC and IgG4‐RD, the serum metabolic profile of PSC has been described previously in comparison to patients with cirrhosis and HCs^19^ using mass spectrometry. Despite the inherent methodological differences between NMR and mass spectrometry that make direct comparisons difficult, some similarities between the results obtained in this study and previous studies were observed. As in our study, serum lactate and 3‐hydroxybutyric acid were unchanged in PSC compared to HCs, whereas glucose levels and mobile choline‐based phospholipids were significantly reduced in patients with PSC. In our study, mobile choline was further reduced in patients with IgG4‐RD compared to PSC. A reduction in choline phospholipids has also been previously identified using mass spectrometry as a key metabolite in IgG4‐RD, distinct from HCs.^21^ The observed reduction in comparison to HCs may be related to the longstanding chronic inflammation common to both diseases, as choline‐based phospholipids involved in cellular structure undergo significant alterations following inflammatory stress, including a decrease in phosphatidylcholines.^22^

More broadly, the key metabolites distinguishing IgG4‐RD from PSC may represent an increased systemic inflammatory phenotype in those with IgG4‐RD, including IgG4‐SC. When previously comparing patients with ulcerative colitis with high and low disease activity, increased blood glucose and lactate and decreased ‐CH_3_ lipoprotein were observed in those with higher disease activity according to both endoscopic and histological scores.^8^ Here, lactate was the top metabolite distinguishing IgG4‐RD, and particularly IgG4‐SC, from PSC. The activation of immune cells during inflammation corresponds to a metabolic switch from resting, aerobic phosphorylation to an effector state, whereby metabolism occurs primarily through aerobic glycolysis.^23^ During this process, lactate is one of the most enriched metabolites of cellular metabolism in inflamed tissues and is now thought to promote T‐cell‐mediated inflammation in chronic inflammatory diseases by inhibiting CD4+ and CD8+ T‐cell motility, thereby retaining a pro‐inflammatory environment in the inflamed tissue.^24^, ^25^ The increase in cell proliferation required to maintain a systemic pro‐inflammatory phenotype may be responsible for the increase in energy intermediates such as glucose and glutamine observed in IgG4‐RD compared to PSC.^23^ Similarly, plasma levels of higher density lipoproteins are often observed to be decreased in chronic inflammation. Levels of high‐density lipoprotein were observed to be decreased in autoimmune diseases such as Crohn’s disease,^26^ systemic lupus erythematosus^27^ and Sjögren's syndrome,^28^ and further research is warranted to determine which specific lipoprotein subclasses mediated the decrease in ‐CH_3_ lipoprotein observed in this study.

In this study, the levels of 3‐hydroxybutyric acid were reduced in patients with IgG4‐RD, compared to patients with PSC and HC. Given that BHB may mediate the resolution of inflammation by inhibiting NLRP3 inflammasome activity,^29^ and that inflammasome activity contributes to the molecular pathophysiology of many autoimmune diseases including IgG4‐RD,^30^ it would be interesting to determine whether the low serum BHB observed in patients with IgG4‐RD is a cause or consequence of disease progression in future prospective studies.

Alongside inflammation, the serum metabolic signature of IgG‐RD (compared to PSC) is reminiscent of previous comparisons between antibody and non‐antibody‐mediated chronic inflammatory diseases. We previously compared the blood metabolome of patients with antibody‐mediated neuromyelitis optica spectrum disorder and relapsing‐remitting multiple sclerosis, which present with similar symptoms and clinical signs.^14^ As in IgG4‐RD, the study revealed an increase in lactate and a decrease in the mobile choline resonance in the antibody‐mediated disease. This may reflect the demand for energy metabolites during chronic (auto)immune activation because of increases in glycolysis and cell proliferation.^31^, ^32^ Indeed, B‐cell depletion therapies are an effective therapy for antibody‐mediated NMOSD, MS and IgG4‐RD suggesting that shared biochemical alterations in immune cell proliferation pathways may be present across these three diseases, as demonstrated with the metabolite changes identified above.

The faecal microbiome and metabolome have been compared between IgG4‐SC and PSC.^20^ Interestingly, whilst many microbial and faecal metabolic features were shared between PSC and IgG4‐SC compared to HCs, IgG4‐SC showed an increase in immune‐related metabolites as determined by mass spectrometry. For example pro‐inflammatory mediators such as arachidonic acid and sphinganine, the precursor of ceramide, were uniquely elevated in the faeces of patients with IgG4‐SC compared to patients with PSC or HCs.^20^ Ceramide synthesis is thought to mediate NLRP3 inflammasome activity,^33^, ^34^, ^35^ whilst downstream metabolism of arachidonic acid by cyclooxygenases leads to the formation of the pro‐inflammatory mediator thromboxane and prostaglandins.^36^ This relates to how the serum metabolic signature of IgG4‐RD and IgG4‐SC in our study resembled a signature of increased systemic inflammation compared to PSC. Despite the former study integrating both 16S microbiome sequencing and faecal metabolite data, PSC and IgG4‐SC were discriminated with an AUC of 0.80, with the most optimal discriminatory model emerging from the faecal metabolome alone (AUC = 0.82). This suggests that the serum metabolome analysed in the present study represents the best approach to classify IgG4‐SC and PSC using non‐invasive, antibody‐independent methods (AUC = 0.95 [95%CI 0.91‐0.99]).

A limitation of our study was the relatively small sample size of patients with IgG4‐SC. IgG4‐SC is a rare condition and for which Oxford is a tertiary referral centre, although it should be noted that, despite this limitation, model performance was determined on independent test data in all cases. While this study is the first to provide proof‐of‐concept that serum metabolomics, in particular relative serum levels of 3‐hydroxybutyric acid, lactate, glutamine and mobile choline, can be used to diagnose IgG4‐SC from PSC, this should be reproduced in a future study. In addition, future work will determine whether these biochemical changes are detectable in the earliest stages of disease, in a prospective cohort of patients. Additionally, although our data corroborate previous findings that suggest a stronger systemic inflammatory metabolic signature associated with IgG4‐RD compared to PSC, functional studies are warranted to investigate the underlying mechanisms. Importantly, however, we show that the serum metabolic signature of IgG4‐RD can be detected from PSC independent of IBD comorbidity, which is much more prevalent in patients with PSC and has been shown to exhibit a strong metabolic signature compared to HCs.^37^

In summary, we show for the first time that blood‐based NMR metabolomics can be used to distinguish IgG4‐SC from PSC, independently of age, sex, medication and comorbid IBD. Accuracy and specificity were improved compared to optimal stratification based on serum IgG4 titre. Future investigation of the disease‐specific metabolite alterations may lead to further insights into the underlying pathophysiology of these conditions and the incorporation of specific serum biomarker panels into clinical practice.

## CONFLICT OF INTEREST

EAS, ELC, DERS, DCA, FP, RP, KDL, MO, JB, KL and AB declare no conflicts of interest. MP is a shareholder in Perspectum, a University of Oxford spin‐out company. AG was employed by UCB Celltech during the preparation of this manuscript and is now an employee of Glaxo Smith Kline (GSK).

## ETHICAL APPROVAL

The study was approved by the Research Ethics Committee, Oxfordshire (ref:10/H0604/51) and (ref:16/YH/0247) and (ref:18/SC/0367) and registered on the NIHR UK portfolio (10776). It was conducted in accordance with the study protocol and the principles of the Declaration of Helsinki (2008), and the International Conference on Harmonization (ICH) Good Clinical Practices standards.

## PATIENT CONSENT STATEMENT

Enrolled study participants provided written informed consent.

## Supporting information


Data S1

## Data Availability

The data that support the findings of this study are available from the corresponding author upon reasonable request.
