# Supplementary material for: A novel serum metabolomic panel distinguishes IgG4‐related sclerosing cholangitis from primary sclerosing cholangitis
Source: Liver Int. 2022 Feb 21;42(6):1344–54. doi: 10.1111/liv.15192 (PMC9546203; doi:10.1111/liv.15192)
Supplement: Supplementary file 1 — Data S1 [file LIV-42-1344-s001.docx]

**Supporting Information**

A novel serum metabolomic panel distinguishes IgG4-related sclerosing cholangitis from primary sclerosing cholangitis

Daniel E. Radford-Smith^1,2^*, Emmanuel A. Selvaraj^3,4,5^*, Rory Peters^3^, Michael Orrell^3^, Jonathan Bolon^3^, Kate Lynch^3^, Daniel C. Anthony^1^, Michael Pavlides^3,4,5^, Alessandra Geremia^3^, Adam Bailey^3,5^, Emma L. Culver^3,5^†, Fay Probert^2^†

*joint first authors

†joint last authors

Affiliations:

^1^Department of Pharmacology, University of Oxford, Oxford, United Kingdom.

^2^Department of Chemistry, University of Oxford, Oxford, United Kingdom.

^3^Translational Gastroenterology Unit, Nuffield Department of Medicine, University of Oxford, United Kingdom.

^4^Oxford Centre for Clinical Magnetic Resonance Research (OCMR), Radcliffe Department of Medicine, University of Oxford, United Kingdom.

^5^NIHR Oxford Biomedical Research Centre, University of Oxford and Oxford University Hospitals NHS Foundation Trust, United Kingdom.

**Supplementary Methods**

**Clinical data collection**

Clinical data were entered onto IgG4-RD Registry database <https://igg4rd.oxnet.nhs.uk> for IgG4-RD and the Oxford ACCESS database for PSC patients. Data on baseline demographics. diagnosis, disease phenotype, distribution of disease, co-morbidities, presence of IBD, medication and treatment history, and serum biochemistry results at the time of blood collection were extracted.

**IgG and IgG4 Nephelometry**

Total serum IgG and IgG4 were measured by nephelometry (BNII analyzer, Siemens, UK). Elevated serum IgG (≥16 g/L) and IgG4 (≥0.86g/L) were defined by institution range (Immunology Department, Churchill Hospital, Oxford). The prozone effect (falsely low serum IgG4 values) was accounted for using serial dilutions where necessary. Serum IgG and subclasses were measured at diagnosis, and comparisons between patients with IgG4-RD and PSC were made before steroid initiation.

**Sample collection, processing, and NMR sample preparation**

Serum samples were collected into two BD Vacutainer® serum separator tubes (SST) that contains a clot activator and serum gel separator. One serum tube was sent to the laboratory for measurement of serum IgG4, bilirubin, alkaline phosphatase (ALP), alanine transferase (ALT) and albumin. The other tube, to be used for NMR spectroscopy, was allowed to stand at room temperature for approximately 30 minutes before being centrifuged at 1,800 x g for 10 minutes at 4°C. The serum supernatant was immediately aliquoted and stored at -80°C until the day of NMR analysis. All samples were processed in this manner within 120 minutes of phlebotomy.

On the day of metabolomics analysis, serum samples were thawed at room temperature and 200 µL was then diluted with 400 μL of 75 mM sodium phosphate buffer prepared in D_2_O (pH 7.4). Samples were briefly centrifuged at 3,000 x g for 5 minutes before transferring to a 5-mm NMR tube.

**NMR spectroscopy and pre-processing.**

All NMR spectra were acquired at 310K using a 700-MHz Bruker AVIII spectrometer operating at 16.4 T equipped with a ^1^H [^13^C/^15^N] TCI cryoprobe (Department of Chemistry, University of Oxford). The noesygppr1d (Bruker, Germany) pulse sequence was used to acquire ^1^H NMR spectra with a 2 s presaturation, 8 data collections, a spectral width of 16 ppm, and an acquisition time of 1.46 s. A 1D ^1^H Carr-Purcell-Meiboom-Gill (CPMG) pulse sequence was used to suppress broad signals (40ms total effective filter time, 32 data collections, acquisition time of 1.5s, relaxation delay of 2s, fixed receiver gain). Diagnostic classes (PSC, IgG4-SC, and IgG-RD) were randomised throughout the run and quality control samples were included at equal intervals to confirm minimal variation.

All spectra were preprocessed in Topspin 3.6 (Bruker, Germany); zero filled by a factor of 2 and multiplied by a 1D exponential corresponding to a 0.3 Hz line broadening. Spectra were baseline corrected with a fifth-degree polynomial and referenced to the lactate doublet at 1.33 ppm. Following visual inspection of all spectra (to identify aberrant baseline correction, referencing, spectral distortion, or contamination) the processed spectra were exported to ACD/Labs Spectrus Processor Academic Edition 12.01 (Advanced Chemistry Development, Inc., Toronto, Canada). Regions of the spectra between 0.83–8.47 ppm were split into 0.02-ppm-wide bins. The residual water resonance region (4.13-5.22 ppm) was removed. The integral of each spectral bin was calculated for statistical analysis and normalised to the sum of the total spectral integrals. Metabolites were assigned using literature values[1], the Human Metabolome Database[2] and via 2D total correlation spectroscopy (TOCSY) experiments.


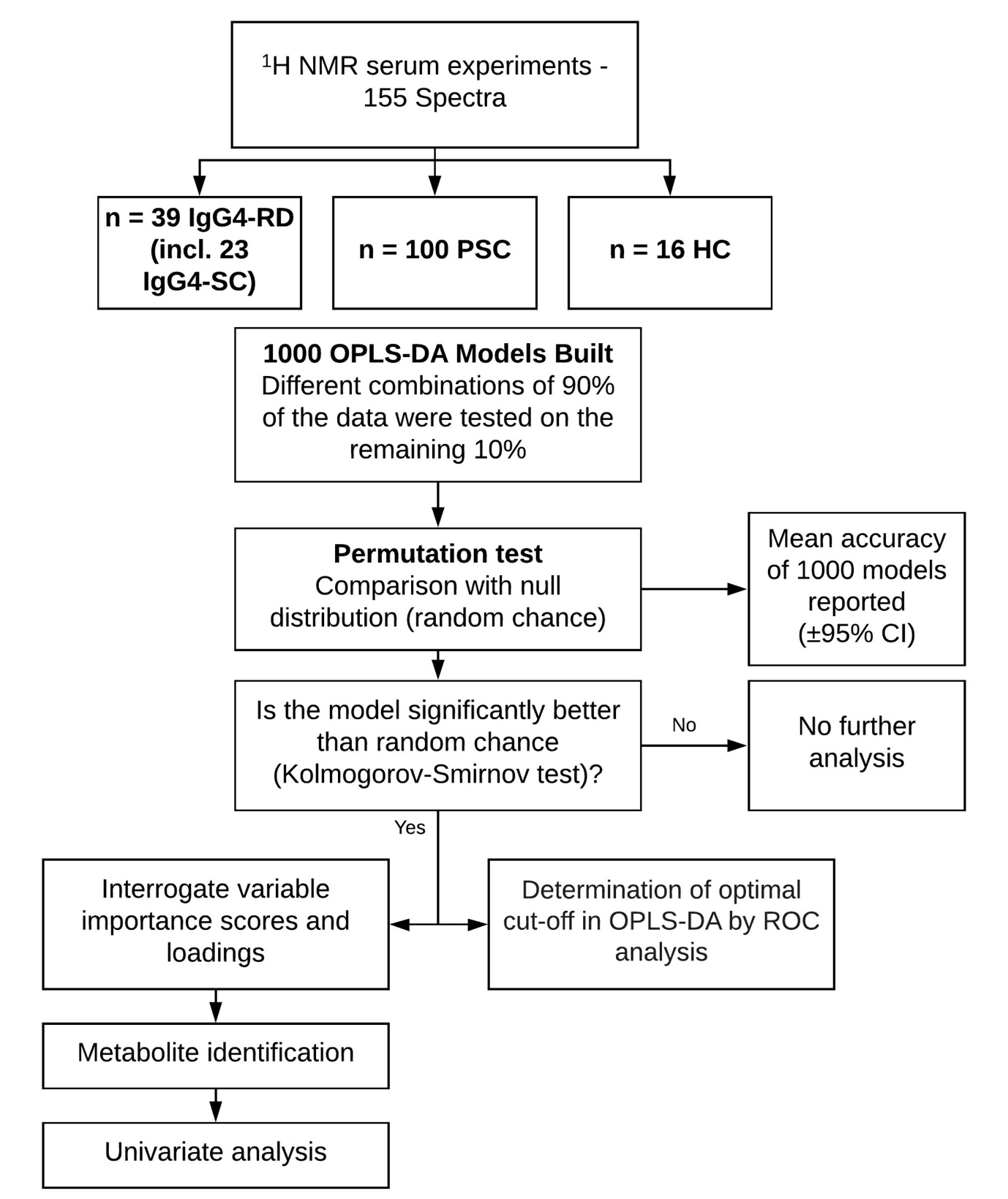


**Figure S1: OPLS-DA methods flowchart.**

**Table S1: Baseline biochemical characteristics of patients with PSC and IgG4-RD in comparison with HCs.**

|  | PSC (n=100) | IgG4-RD (n=39) | HC (n=16) | p-value |
| --- | --- | --- | --- | --- |
| ^†^Age, years | 45.0±19.0 (12-84) | 64.9±12.6 (33-83) | 35.4±6.5 (25-52) | **p<0.0001** |
| Sex (% female) | 38.0 | 15.8 | 37.5 | p=0.24 |
| ^#^ALT, IU/L | 38.5, 23.8-82.8 (3-2683) | 25.0, 17.0-41.8 (8-1034) | 21, 14.3-29.3 (7-31) | p=0.99 |
| ^#^ALP, IU/L | 210.5, 113.0-372.0 (42-1316) | 157.0, 112.3-208.8 (33-578) | 55.0, 46.3-71.5 (31-93) | **p<0.0001** |
| ^†^Albumin, g/L | 38.52±5.58 (23-50) | 40.29±5.32 (26-49) | 43.1±2.18 (40-48) | **p=0.0024** |
| ^#^Bilirubin, µmol/L | 10.5, 8.0-20.0 (3-286) | 9.0, 8.0-16.0 (4-203) | 10.5, 8.0-13.0 (4-21) | p=0.24 |
| ^#^Median, IQR (range). ^†^Mean±SD (range). PSC, primary sclerosing cholangitis; IgG4-RD, IgG4-sclerosing cholangitis, IBD, inflammatory bowel disease; UDCA, ursodeoxycholic acid; ALT, alanine aminotransferase; ALP, alkaline phosphatase. Brown-Forsythe and Welch ANOVA with Dunnet’s T3 post-hoc comparisons or chi-squared test. | | | | |


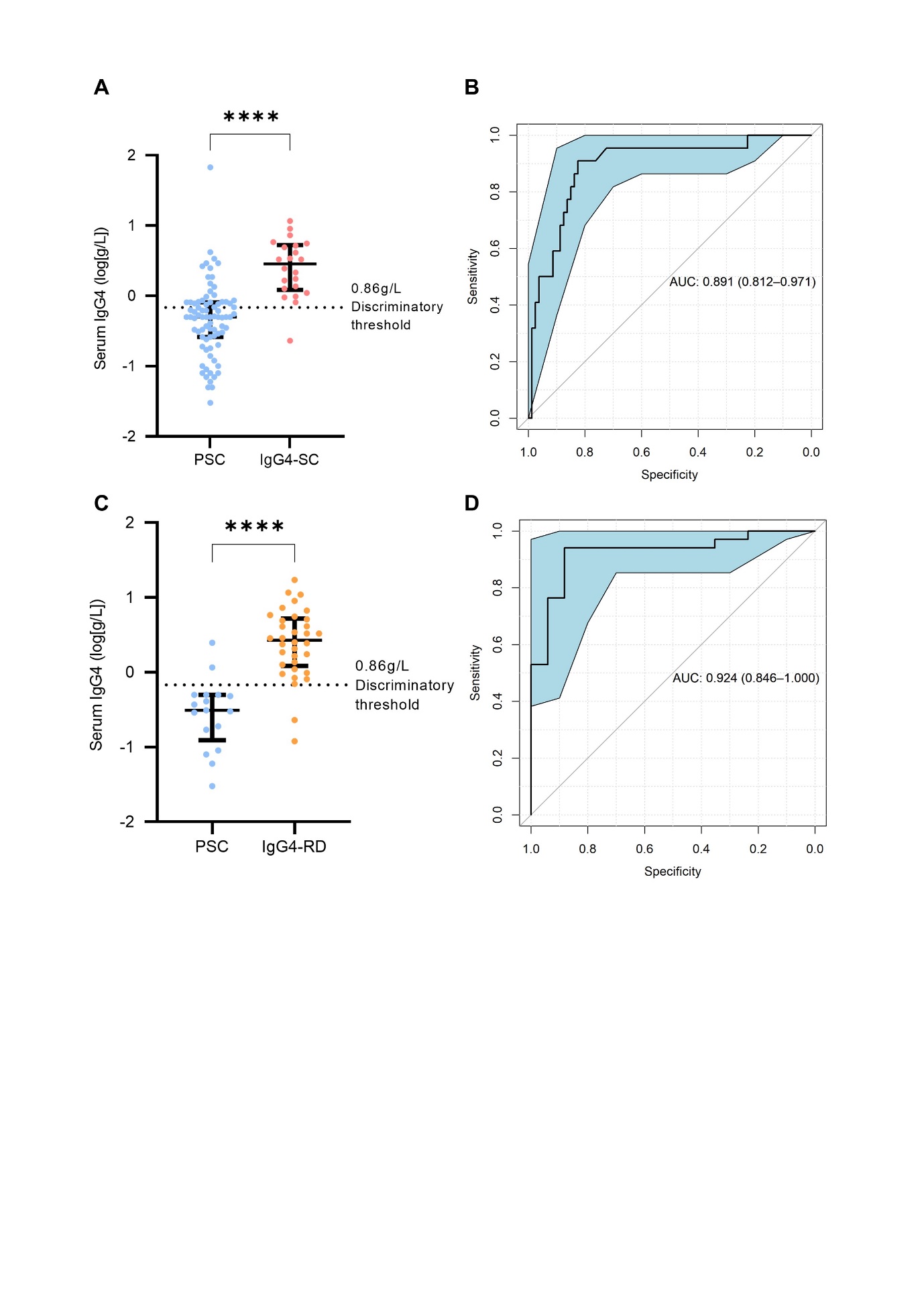
**Figure S2: Serum IgG4 titre accurately distinguishes IgG4 hepatopathy from PSC, particularly when patients with IBD are excluded.** Patients with **A)** IgG4-SC (n = 22) and **C)** IgG4 disease excluding IBD (n = 33) had elevated serum IgG4 antibodies compared to PSC (n=80) and PSC excluding IBD (n = 17) respectively (Kolmogorov-Smirnov, both p < 0.0001). Median ± interquartile range showing all points. **B)** Receiver-operating characteristic (ROC) curve for IgG4 titre classifying PSC v. IgG4-SC and **D)** PSC excluding IBD v. IgG4 disease excluding IBD. ROC curves show AUC ± 95% confidence intervals.


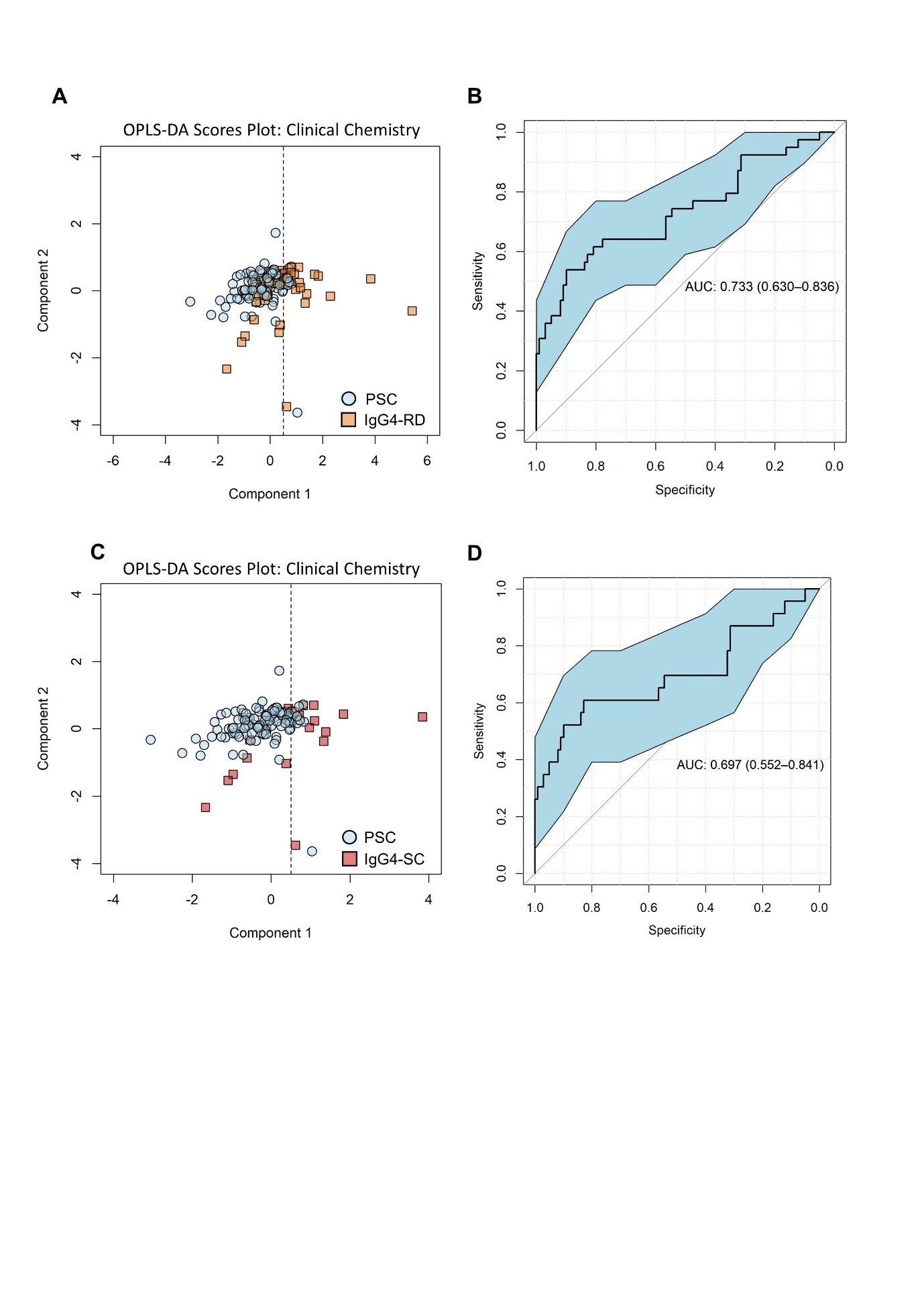


**Figure S3: Multivariate OPLS-DA analysis of routine serum clinical chemistry measures does not afford an improved diagnostic specificity for discriminating IgG4 disease from PSC.** OPLS-DA scores plot of **A)** PSC (n=80) v. IgG4-RD (n=38) and **C)** PSC (n=80) v. IgG4-SC (n=22) using clinical chemistry parameters including IgG4 titre. ROC curve classifying **B)** PSC v. IgG4-RD and **D)** PSC v. IgG4-SC. ROC curves show AUC ± 95% confidence intervals. Clinical chemistry variables included in the OPLS-DA and ROC analysis were serum IgG4 titre, IgG titre, ALT, ALP, albumin, and bilirubin.


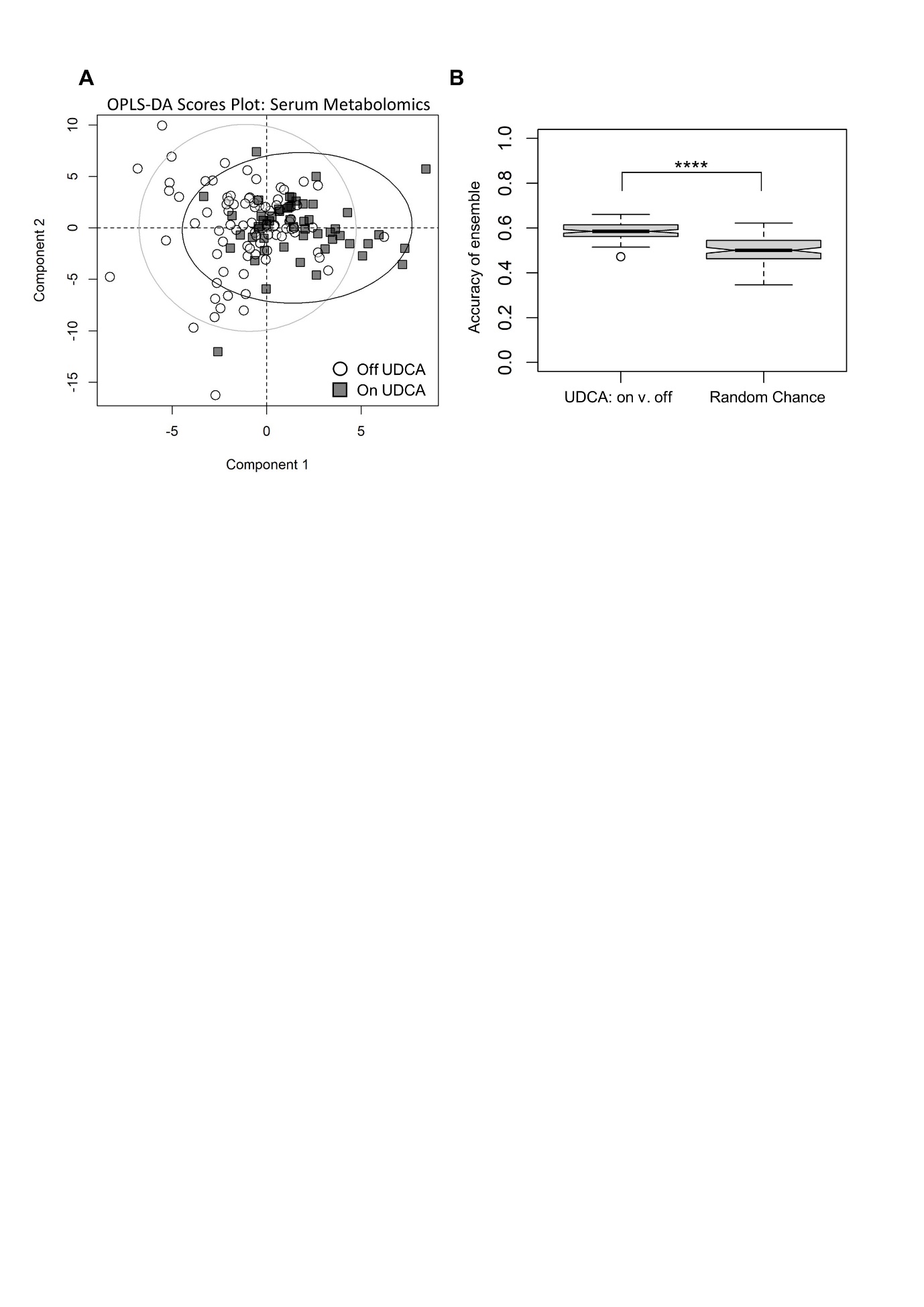


**Figure S4: Ursodeoxycholic acid (UDCA) treatment does not account for the serum metabolic differences between individuals with PSC and IgG4. A)** OPLS-DA scores plot of individuals treated vs. untreated with UDCA, independent of disease classification. **B)** Average accuracy of ensemble of OPLS-DA models compared to random chance (Kolmogorov-Smirnov test, p < 0.0001 over n = 1000 models). Boxplot showing 95% confidence interval.


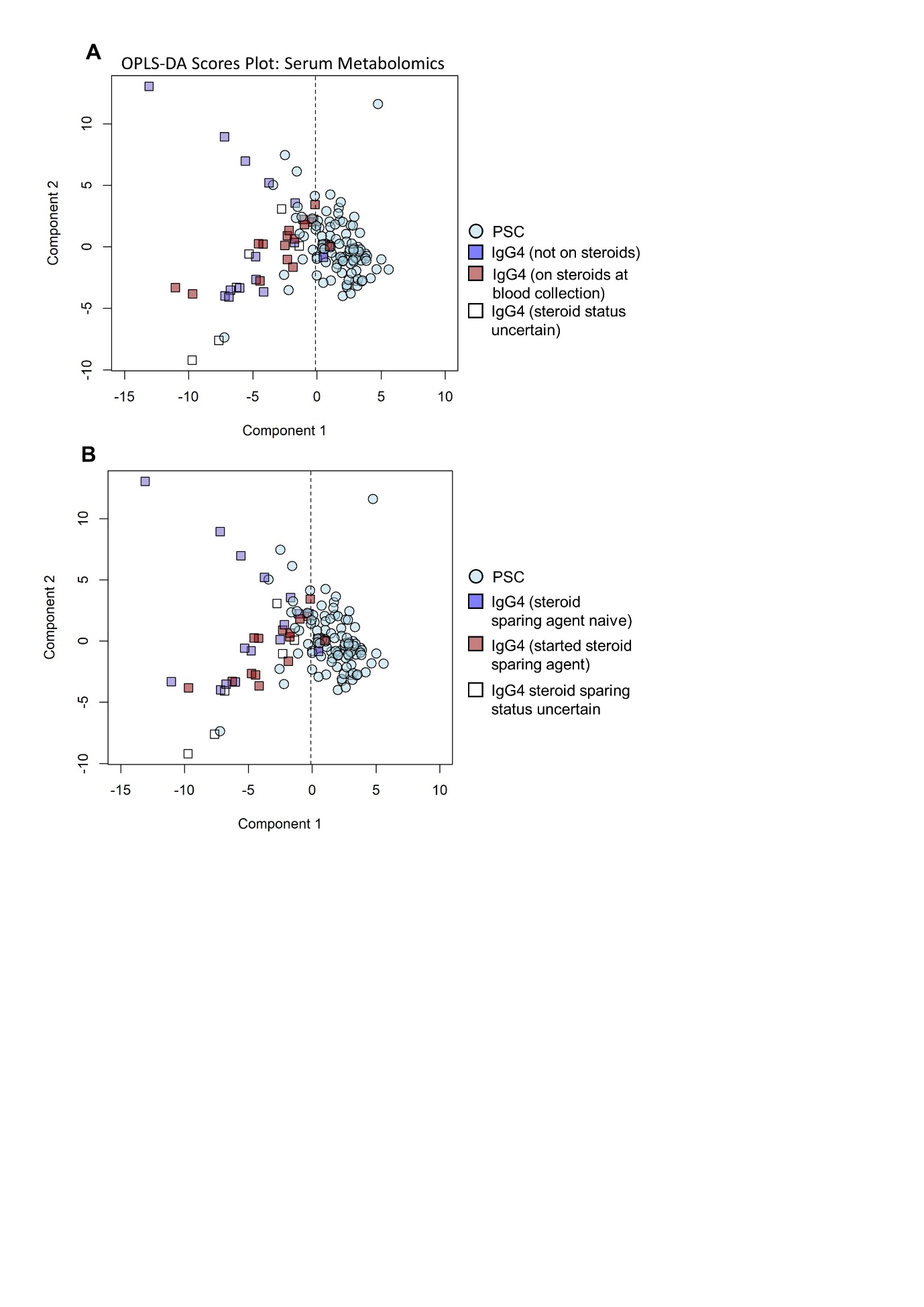
**Figure S5: Treatment status of patients with IgG4 disease does not interfere with the metabolic signature of disease.** OPLS-DA scores plots of PSC (n = 100) v. IgG4 disease (n = 39) using serum metabolomic data, coloured by **A)** steroid treatment at time of blood collection or **B)** steroid-sparing agent treatment at time of blood collection. PSC patients were naïve to these treatments.


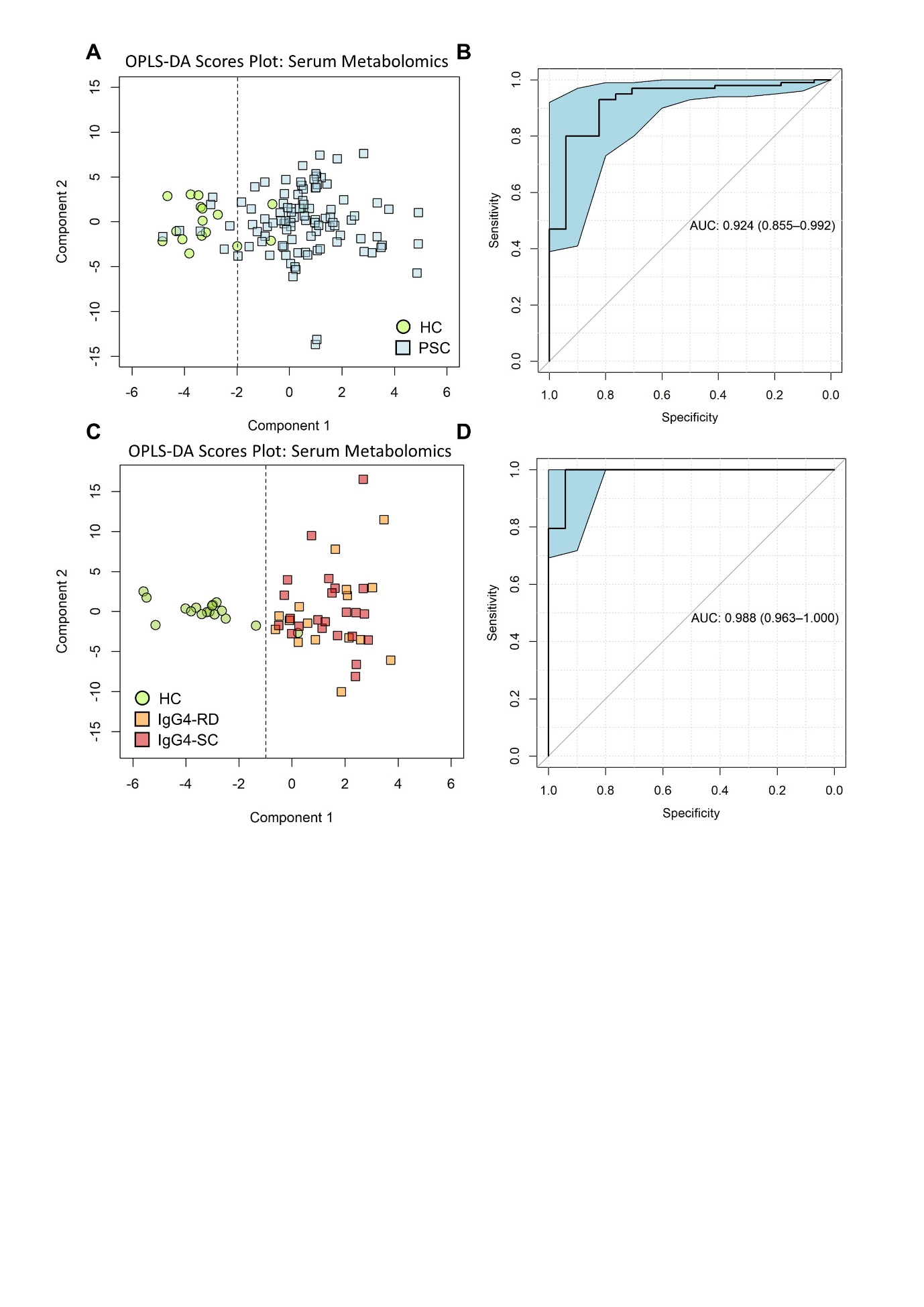
**Figure S6: The metabolic signatures of PSC and IgG4 disease are independently distinct from that of healthy controls (HC).** OPLS-DA scores plot of **A)** HC (n = 16) v. PSC (n = 100) and **C)** HC v. IgG4 disease (n = 39) using serum metabolomic data. **B)** ROC curve classifying HC v. PSC and **D)** classifying HC v. IgG4-related disease (n = 39). ROC curves show AUC ± 95% confidence intervals.


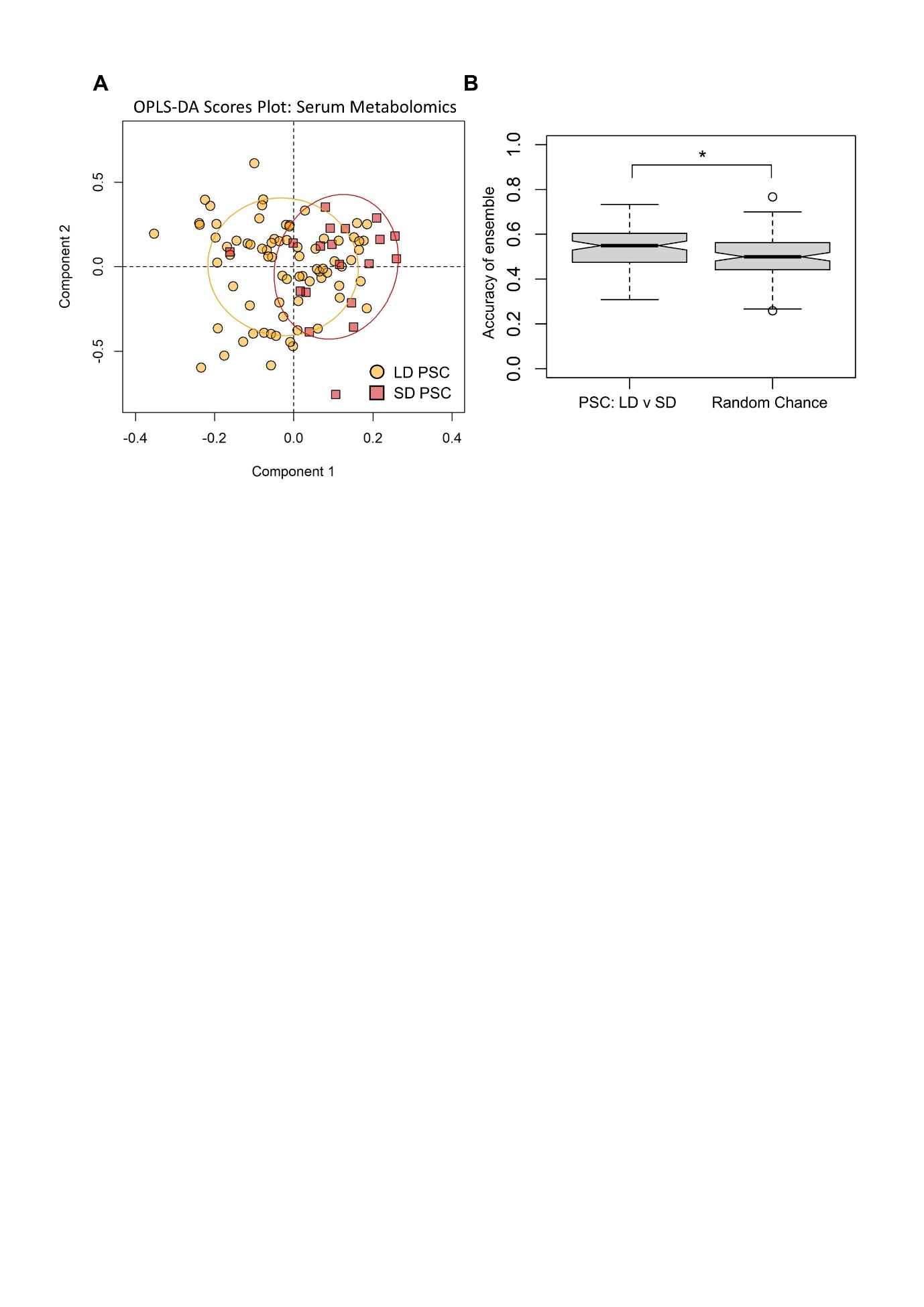


**Figure S7: OPLS-DA of the serum metabolome distinguished large-duct and small-duct PSC with an average accuracy of 54.3%. A)** OPLS-DA scores plot of individuals with large-duct and small-duct PSC. **B)** Average accuracy of ensemble of OPLS-DA models compared to random chance (Kolmogorov-Smirnov test, p = 0.012 over n = 1000 models). Boxplot showing 95% confidence interval.

**Table S2. Confusion matrix IgG4 titre.**

|  | Reference | | |
| --- | --- | --- | --- |
| Prediction | **PSC** | **IgG4-RD (all)** | **IgG4-SC** |
| **PSC** | 66 | 6 | 2 |
| **IgG4-RD (all)** | 14 | 32 | - |
| **IgG4-SC** | 14 | - | 20 |

**Table S3. Confusion matrix serum metabolomics.**

|  | Reference | | |
| --- | --- | --- | --- |
| Prediction | **PSC** | **IgG4-RD (all)** | **IgG4-SC** |
| **PSC** | 72 | 8 | 8 |
| **IgG4-RD (all)** | 4 | 34 | - |
| **IgG4-SC** | 2 | - | 20 |

**References**

1 Lenz EM, Bright J, Wilson ID, Morgan SR, Nash AF. A 1H NMR-based metabonomic study of urine and plasma samples obtained from healthy human subjects. J Pharm Biomed Anal 2003;**33**:1103-15.

2 Chong J, Soufan O, Li C, Caraus I, Li S, Bourque G*, et al.* MetaboAnalyst 4.0: towards more transparent and integrative metabolomics analysis. Nucleic Acids Res 2018;**46**:W486-W94.
